# Progress Toward Polio Eradication — Worldwide, 2013–2014

**Published:** 2014-05-30

**Authors:** Edna K. Moturi, Kimberly A. Porter, Steven G.F. Wassilak, Rudolf H. Tangermann, Ousmane M. Diop, Cara C. Burns, Hamid Jafari

**Affiliations:** 1 EIS officer, CDC; 2 Global Immunization Division, Center for Global Health, CDC; 3 Polio Eradication Department, World Health Organization; 4 Division of Viral Diseases, National Center for Immunization and Respiratory Diseases, CDC

In 1988, the World Health Assembly of the World Health Organization (WHO) resolved to interrupt wild poliovirus (WPV) transmission worldwide, and in 2012, the World Health Assembly declared the completion of global polio eradication a programmatic emergency for public health (1). By 2013, the annual number of WPV cases had decreased by >99% since 1988, and only three countries remained that had never interrupted WPV transmission: Afghanistan, Nigeria, and Pakistan. This report summarizes global progress toward polio eradication during 2013–2014 and updates previous reports (2). In 2013, a total of 416 WPV cases were reported globally from eight countries, an 86% increase from the 223 WPV cases reported from five countries in 2012 (3). This upsurge in 2013 was caused by a 60% increase in WPV cases detected in Pakistan, and by outbreaks in five previously polio-free countries resulting from international spread of WPV. In 2014, as of May 20, a total of 82 WPV cases had been reported worldwide, compared with 34 cases during the same period in 2013. Polio cases caused by circulating vaccine-derived poliovirus (cVDPV) were detected in eight countries in 2013 and in two countries so far in 2014 (4). To achieve polio eradication in the near future, further efforts are needed to 1) address health worker safety concerns in areas of armed conflict in priority countries, 2) to prevent further spread of WPV and new outbreaks after importation into polio-free countries, and 3) to strengthen surveillance globally. Based on the international spread of WPV to date in 2014, the WHO Director General has issued temporary recommendations to reduce further international exportation of WPV through vaccination of persons traveling from currently polio-affected countries (5).

## Routine Vaccination Coverage

During 2012, the latest year for which complete data are available, global coverage of infants by age 12 months with 3 doses of polio vaccine (Pol3) through routine vaccination was estimated at 84%. Pol3 coverage estimates by WHO region were 77% in the African Region (AFR), 74% in the South-East Asia Region (SEAR), 82% in the Eastern Mediterranean Region (EMR), 93% in the Region of the Americas (AMR), 96% in the European Region (EUR), and 97% in the Western Pacific Region (WPR). Among the countries where polio is endemic, estimated national Pol3 coverage was 59% in Nigeria, 71% in Afghanistan, and 75% in Pakistan. However, substantial variability in coverage exists within these countries (6).

## Supplementary Immunization Activities

In 2013, 265 supplementary immunization activities (SIAs) using oral poliovirus vaccine (OPV) were conducted in 42 countries, 52% (137) in AFR and 45% (118) in EMR. These included 113 national immunization days, 134 subnational immunization days, 13 child health days, and five large-scale mop-up rounds. About 2.24 billion doses of OPV were administered over the year to a target population of mostly children aged <5 years; of these doses, 995 million were trivalent, 1.2 billion were bivalent (types 1 and 3) and 8 million were type 1 monovalent OPV. Short-interval additional dose SIAs (7) were implemented in Afghanistan to boost population immunity using monovalent OPV and/or bivalent OPV in hard-to-reach areas. An extensive set of outbreak response and preventive SIAs have been planned and conducted in the Middle East to respond to the WPV type 1 (WPV1) outbreak in Syria (Table 1).

## Poliovirus Surveillance

Polio cases caused by WPV or by cVDPV are detected through surveillance for acute flaccid paralysis (AFP) cases and testing of stool specimens at WHO-accredited laboratories of the Global Polio Laboratory Network (8). Of the 12 countries reporting WPV and/or cVDPV cases during 2012–2013, the two main AFP surveillance performance indicators* were met at the national level in five countries (42%). All EMR countries met surveillance performance indicators, except Syria. Surveillance quality indicators in several high-risk countries with recent outbreaks deteriorated during 2013, compared with 2012. In only four (33%) of the 12 countries polio-affected during 2012–2013 was ≥80% of the population living in sub-national areas where both indicators were met during 2013 (Afghanistan, Nigeria, Pakistan, and Somalia). Despite this, virologic evidence showed surveillance gaps in each of these four countries (8).

## Reported WPV Cases

All 416 WPV cases reported in 2013 were WPV1. Of these, 22% were cases detected in Pakistan and 62% were cases in new outbreaks after WPV1 importations into previously polio-free countries. As of May 20, during January–April 2014, the low transmission season for poliovirus, 82 WPV1 cases were reported globally from eight countries, an increase from 34 WPV1 cases reported from three countries during the same period in 2013 (Table 2). During 2014, as of May 20, WPV1 has already spread internationally from three countries: in central Asia (from Pakistan to Afghanistan), the Middle East (Syria to Iraq), and in Central Africa (Cameroon to Equatorial Guinea). WPV type 3 (WPV3) cases have not been detected in Pakistan since April 2012 and in Nigeria since November 2012. No WPV type 2 cases have been detected anywhere in the world since 1999.

### Afghanistan

In 2013, a total of 14 WPV1 cases were reported in 10 districts, a 62% decrease from 37 cases reported in 21 districts in 2012 and a 52% decrease in the number of affected districts. This is the lowest level of reported WPV since 2004; all but one case in 2013 and all cases in 2014 were reported from eastern Afghanistan and genetically linked to WPV importation from Pakistan. In 2013, only one case was reported from Helmand Province in southern Afghanistan, which had been the main region of Afghanistan where polio was endemic up to 2012. During January–April 2014, four WPV1 cases were reported, compared with two cases reported during the same period in 2013.

### Nigeria

In 2013, 53 WPV1 cases were reported in 30 districts, a 57% reduction from the 122 WPV cases (109 WPV1 and 19 WPV3 cases) in 60 districts reported in 2012, and a 50% decrease in the number of affected districts. During January–April 2014, Nigeria reported three WPV1 cases, an 88% decrease compared with 16 cases reported during the same period in 2013. In 2013, SIAs were suspended temporarily in some areas of armed conflict in northeastern Nigeria.

### Pakistan

In 2013, 93 WPV1 cases were reported in 23 districts, a 60% increase from 58 WPV cases (55 WPV1 cases, two WPV3 cases, and one case with WPV1 and WPV3 coinfection) in 28 districts in 2012, and an 18% reduction in the number of affected districts. During January–April 2014, Pakistan reported 66 cases, compared with eight cases reported during the same period in 2013. Since mid-2012, local authorities have imposed a complete ban on conducting SIAs in North Waziristan, South Waziristan, and one part of northwestern Pakistan, and in 2013, SIAs were suspended temporarily in some areas of Pakistan because of risks for violence.

## Countries with Polio Outbreaks

The number of WPV cases in outbreaks after WPV importation into previously polio-free countries increased from six cases in two countries (Chad and Niger) in 2012 to 256 cases in five countries in 2013 (Figure). Importation of WPV1 from Nigeria into the Horn of Africa resulted in 217 cases in 2013 (nine in Ethiopia, 14 in Kenya, and 194 Somalia); one outbreak case was reported by Ethiopia in 2014. Importation from Pakistan into Syria resulted in 35 cases in 2013 and one case in 2014; in 2014, a WPV case in Iraq resulted from WPV imported from Syria. Four WPV1 cases were reported in Cameroon in 2013 and three in 2014, and three cases were reported in Equatorial Guinea in the first quarter of 2014 (Table 2). On genomic sequence analysis, the isolates were of Nigerian origin most closely linked with WPV cases reported from Chad in 2012.

### Discussion

Despite increases in cases since 2012, substantial progress toward polio eradication has occurred. No WPV3 case has been identified globally since November 2012 in Nigeria, raising the possibility that WPV3 transmission may have been interrupted globally. In March 2014, the WHO SEAR joined the WHO AMR, WPR and EUR as being certified free of indigenous wild poliovirus. With this achievement, 80% of the world’s population now lives in WHO regions certified as polio-free. Indigenous WPV transmission within AFR and EMR, the two remaining WHO regions where polio is endemic, is now restricted to fewer geographical areas within each of the three remaining countries where polio is endemic than ever before. The decrease in the number of reported WPV cases and number of affected states and districts in Nigeria was associated with significantly improved SIA quality indicators during late 2012 and early 2013 (9). Current WPV transmission in Nigeria appears to be restricted to Kano and Borno states, although gaps in surveillance quality remain.

During 2010–2012, the conflict in Afghanistan prevented vaccinators from safely accessing children in many areas of the southern region of Afghanistan. However, systematic negotiations greatly improved access to children in 2013, which, together with successful efforts to improve the quality of SIAs, substantially reduced transmission of endemic WPV (7). However, the success of global polio eradication is being challenged by major limitations in access and physical security within other countries.

In Pakistan, targeted attacks against polio workers and police officers assigned to protect them have seriously compromised the implementation of SIAs in parts of the Federally Administered Tribal Areas, Khyber Pakhtunkhwa province, and Karachi city. The continued ban on polio vaccination in North and South Waziristan, Federally Administered Tribal Areas where local leaders have prevented vaccination of >350,000 children since June 2012, is largely responsible for the increase in WPV cases in 2013 and 2014 in Pakistan and for recent WPV importation into Afghanistan and war-torn Syria. However, as of the end of April, 12 consecutive SIAs were carried out in 2014 already in Khyber Pakhtunkhwa province, demonstrating strong political commitment and engagement of local communities, religious leaders, and humanitarian organizations to reach unvaccinated children in these areas (10).

Terrorist acts by antigovernment elements in Nigeria have prevented vaccinators from visiting some areas of Borno state since early 2013; however, vaccination access has gradually improved, and 84% of children were accessible by March 2014.†

Limitations in access and physical security have also greatly affected the ability to promptly control and end outbreaks. Outbreak control has also been compromised by suboptimal SIA implementation, and incomplete understanding of outbreak dynamics resulting from variable AFP surveillance quality. The outbreak in the Horn of Africa has lasted >9 months after initial confirmation, partly caused by limitations in the quality of outbreak response in parts of Somalia not under government control and difficult-to-reach areas within Ethiopia. The ongoing circulation of WPV1 in Cameroon and Equatorial Guinea poses a risk for wider spread, including into populations affected by ongoing civil unrest in the Central African Republic; an aggressive outbreak response is being planned to include neighboring countries to limit further extension of transmission.

With further restriction of the geographic extent of WPV circulation in the countries where polio is endemic, and provided that outbreaks after importation into polio-free countries can be prevented or interrupted promptly, interruption of global transmission could be achieved in the near future. The GPEI has developed the Polio Eradication and Endgame Strategic Plan for 2013–2018§ to 1) interrupt all poliovirus transmission, 2) progressively withdraw OPV and introduce inactivated poliovirus vaccine, 3) certify polio eradication, and 4) transition assets and infrastructure to routine immunization programs as part of GPEI legacy.

The Director General of WHO has declared the recent international spread of WPV a public health emergency of international concern (5) and issued temporary recommendations under the International Health Regulations (IHR 2005) to reduce international exportation of WPV through 1) ensuring that residents and long-term visitors traveling from Cameroon, Pakistan, and Syria receive vaccination before international travel, and 2) encouraging residents and long-term visitors traveling from Afghanistan, Equatorial Guinea, Ethiopia, Iraq, Israel, Somalia, and Nigeria to receive vaccination before international travel and 3) ensuring that such travelers are provided an International Certificate of Vaccination documenting vaccination status.¶ At this stage in the GPEI, enhanced commitment by countries and GPEI partners in a coordinated international effort is crucial to maintaining current gains and to complete polio eradication.

What is already known on this topic?

The Global Polio Eradication Initiative (GPEI) began in 1988. Wild poliovirus (WPV) transmission has decreased by >99% since then, and currently WPV transmission remains endemic only in Afghanistan, Nigeria, and Pakistan. Outbreaks caused by importation of WPV cases have been detected in previously polio-free countries, and in 2012, the World Health Assembly of the World Health Organization (WHO) declared the completion of polio eradication a global public health emergency.

What is added by this report?

Significant progress toward polio eradication has been achieved during 2012–2013; the WHO South-East Asia Region was certified polio-free, the geographic extent of WPV transmission has decreased within the countries where polio is endemic, and there is possible eradication of WPV type 3. An increase in the number of polio cases occurred because of ongoing outbreaks in Pakistan and outbreaks resulting from international spread of polioviruses into the Horn of Africa and the Middle East. Threats of physical violence in areas of conflict have emerged as a major risk for polio eradication efforts.

What are the implications for public health practice?

Current GPEI progress indicates that polio eradication is achievable. However, the occurrence of outbreaks in previously polio-free countries demonstrates that all countries and regions remain at risk so long as WPV transmission continues in any country. Strengthened AFP surveillance performance and improved supplementary immunization activity quality might prevent or further limit the spread of WPV.

## Figures and Tables

**FIGURE:**
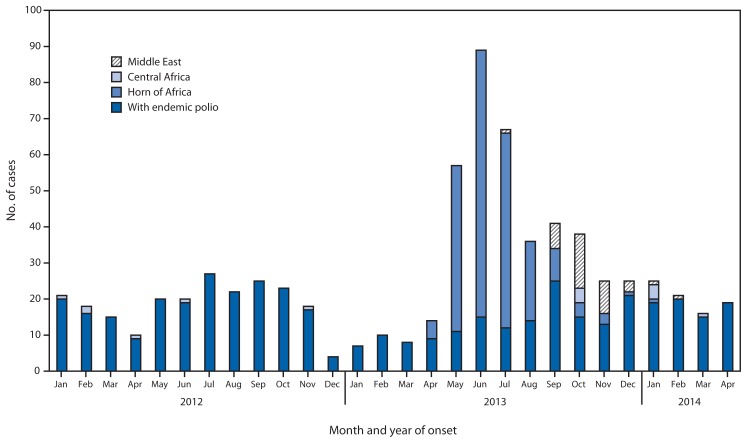
Number of wild poliovirus cases among countries with endemic polio and regions with recent polio outbreaks, by month and year of onset — January 2012–April 2014* ^*^ Data as of May 20, 2014.

**TABLE 1:** Number of supplemental immunization activities (SIAs) conducted and number of oral poliovirus vaccine doses administered, by country/area — worldwide, 2013 and 2014

	2013		2014	
		
Country/Area	SIAs	Doses	SIAs	Doses
Afghanistan	19	37,410,609	16	36,783,744
Angola	3	14,769,565	1	7,583,041
Bangladesh	1	30,105,022		
Benin	4	3,808,701	2	3,911,445
Burkina Faso	5	9,326,642	2	14,943,494
Cameroon	8	10,489,620	8	17,939,541
Central African Republic	4	1,273,793	3	1,047,098
Chad	13	18,837,112	3	6,633,913
Cote d’Ivoire	3	18,195,078	1	8,836,776
Democratic Republic of the Congo	7	32,591,315	6	20,713,377
Djibouti	3	302,453		
Egypt	5	38,670,031	1	15,596,691
Equatorial Guinea			4	545,015
Eritrea	2	692,235		
Ethiopia	13	34,460,757	5	17,827,430
Gabon			1	382,904
Gambia	2	507,025		
Ghana	2	6,119,545		
Guinea	3	7,508,602	1	3,932,186
Guinea-Bissau	2	345,067		
India	6	320,043,470	5	567,008,989
Iran	4	2,320,111	4	1,241,781
Iraq	8	23,579,384	4	16,191,343
Jordan	5	2,907,026	1	1,117,898
Kenya	15	28,025,788	6	26,738,433
Laos	1	361,446		
Lebanon	3	996,160	2	856,179
Liberia	3	1,128,688		
Mali	6	20,779,108	2	15,838,686
Mauritania	2	769,707		
Nepal			1	5,786,332
Niger	10	22,724,996	3	16,807,958
Nigeria	22	379,934,093	11	200,698,979
Pakistan	19	219,575,821	22	171,011,355
Philippines			2	32,827,615
Senegal	2	6,545,177		
Sierra Leone	4	1,716,577		
Somalia	28	37,473,206	11	10,402,708
South Sudan	6	13,895,568	2	6,913,709
Sudan	4	25,608,309	2	13,106,801
Syria	6	12,369,813	5	9,520,753
Turkey	2	3,118,271	3	1,418,787
Uganda	2	6,434,132		
West Bank and Gaza Strip	1		1	
Yemen	7	29,258,816	1	5,797,919
**Total**	**265**	**1,424,978,839**	**142**	**1,259,962,880**

* Data as of April 29, 2014.

**TABLE 2:** Number of reported wild poliovirus cases, by country and serotype —worldwide, January–April 2013 and 2014*

		January–April	
		
Country	2013	2013	2014
**With endemic polio**			
Afghanistan	14	2	4
Nigeria	53	22	3
Pakistan	93	8	66
**With polio outbreaks**			
Iraq	0	0	1
Equatorial Guinea	0	0	3
Cameroon	4	0	3
Somalia	194	1	0
Syria	35	0	1
Ethiopia	9	0	1
Kenya	14	1	0
Niger	0	0	0
**Total**	**416**	**34**	**82**
**Total endemic**	**160**	**32**	**73**
**Total in outbreak**	**256**	**0**	**9**

* Data as of May 20, 2014.

## Footnotes

* Standard performance indicators include 1) the rate of nonpolio AFP cases (target = ≥1 case per 100,000 population aged <15 years for countries in WHO regions certified as polio-free; all other countries should achieve annual rates of ≥2), and 2) the proportion of AFP cases with adequate stool specimens (target = ≥80%).

† Global Polio Eradication Initiative Status Report. Available at http://www.polioeradication.org/Portals/0/Document/Aboutus/Governance/IMB/10IMBMeeting/2.2_10IMB.pdf.

§ The Polio Eradication and Endgame Strategic Plan 2013–2018, available at http://www.polioeradication.org/Portals/0/Document/Resources/StrategyWork/PEESP_EN_US.pdf, is a comprehensive, long-term strategy that addresses what is needed to deliver a polio-free world by 2018.

¶ Additional information is available at http://wwwnc.cdc.gov/travel.
